# Prevalence of depression and associated factors among patients with epilepsy at the University of Gondar Comprehensive Specialized Hospital, Northwest Ethiopia, 2019

**DOI:** 10.1371/journal.pone.0257942

**Published:** 2021-10-25

**Authors:** Banchlay Addis, Maereg Wolde, Amare Minyihun, Andualem Yalew Aschalew

**Affiliations:** 1 Department of Health Systems and Policy, College of Medicine and Health Science, University of Gondar, Gondar, Ethiopia; 2 Department of Health Education and Behavioral Sciences, College of Medicine and Health Science,University of Gondar, Gondar, Ethiopia; Universita degli Studi di Roma La Sapienza, ITALY

## Abstract

**Introduction:**

Depression is a commonly overwhelming problem among patients with epilepsy which compromises their quality of life especially in developing countries. Previously limited studies were conducted using Becks Depression Inventory tool in Ethiopia. The aim of this study’s objective was to determine the prevalence of depression and associated factors among patients with epilepsy.

**Methods:**

Institution based cross-sectional study was employed at the University of Gondar Comprehensive Specialized Hospital from March 01–30, 2019.A total of 370 participants were selected using an interview administered structured questionnaire. Hospital Anxiety and Depression Scale was used to assess the prevalence of depression.Multivariable logistic regression analysis was done to investigate potential predictors and variables with a P-value of < 0.05 and a 95% confidence interval were considered statistically significant.

**Results:**

A total of 370 study participants participated with a response rate of 92%. From the total respondents 37% experienced depression. Perceived stigma (AOR = 3.89, CI: 2.27, 6.68), educational status (AOR = 0.48, CI: 0.25, 0.92), residence (AOR = 0.5, CI: 0.28, 0.89), frequency of seizure (AOR = 2.07, CI: 1.01, 4.23) and social support (AOR = 2.73, CI: 1.41–5.31) were significantly associated with depression status.

**Conclusion:**

This study revealed that prevalence of depression among Epileptic patients was high. Perceived stigma, educational status, residence, frequency of seizure and social support were significantly associated with depression status. Thus, health care workers better to give more emphasis to patients with perceived stigma, higher number of seizure frequency and to those with poor level of social support.

## Introduction

Epilepsy is a common neurological disorder characterized by recurrent and unpredictable seizures associated with significant psychological and social consequences [1]. It is a global public health problem in the world with approximately 46 million people is estimated to suffer from this public health problem. While epilepsy contributes 1% of the global burden of disease, it accounts 80% in the developing countries [2]. In general, the incidence rate in developing countries was higher than in developed countries, as the blunt incidence of epilepsy in Ethiopia is 64 per 100,000,000 [3]. People with epilepsy (PWE) are more vulnerable to psychiatric disorders, which is 9% higher in PWE than in the general population, and depression rates are 22% higher in the general population [4].

Depression is one of the most frequent comorbidity among patients with epilepsy [1,5,6]. It is characterized by symptoms such as changes in appetite, altered sleep habits, increased or decreased level of activity, diminished focus and concentration, and dramatically reduced feelings of self-worth and extreme types, death desire or suicidal attempts [7].

A bidirectional interaction is thought to be present between epilepsy and depression. But depression does not always follow the onset of epilepsy. It might proceed by the onset of seizure [8,9].

Among the 50 million PWE worldwide, 9.5% to 85% are also likely to suffer from depressive disorders, and more than 80% of them reside in low-income regions where psychiatric comorbidity is often under recognized and undertreated [10].

Some studies show lower prevalence of depression in patients with epilepsy while others report high prevalence depending on the setting. This is higher than patients with other chronic diseases, which is the primary reason for psychiatric hospitalization and psychotropic medications [11].

A systematic study conducted in Sub-Saharan Africa found that the combined prevalence of depression among epilepsy patients was 32.71 percent; while the regional sub-group research showed that the combined prevalence in East Africa was 34.52 percent and 29.69 percent, respectively, in Southern Africa [4].

Regarding its prevalence in Ethiopia 51.2 percent, 48.1 percent, 49.3 percent, at Benchimaji, Mettu and Jimma Hospital, respectively [10,12,13]. Similarly, in Amanuel Specialized Hospital, the instuition-based analysis using PHQ-9 was43.8 percent and HADS instruments were 43.8 percent and 32.8 percent [1,12]. In addition, a study conducted at the University of Gondar Comprehensive Specialized Hospital prior to six years to determine the prevalence of depression among epileptic patients was 45.2% [13].

Evidences from different literature indicate that level of education, frequency of seizure [10,14], income, marital status [10], residence [14], age [8], perceived stigma [14], onset of disease, number of drug [10,12,15], adverse drug event [16], comorbidity [4], drug frequency and social support [10] were associated with depression. Although a study was performed six years ago in this study area with a different instrument (BDI) that has 21 items based on detecting depression, including the normal population. Whereas the HADS instrument is short with 14 items, acceptable for patients and easy to complete in the waiting room of the hospital. The approach explicitly differentiates the definitions of anxiety and depression. Therefore this study aims to asses prevalence of depression and factors with HADS tool.

## Methods

### Study design and study setting

Institution based cross-sectional study was conducted at the University of Gondar Comprehensive Specialized Hospital (UoGCSH) from March 01–30, 2019. The hospital is one of the tertiary-level teaching hospitals in Amhara National Regional State. It serves approximately seven million people and provides inpatient and outpatient services including patients with chronic illnesses. The chronic illness follow-up outpatient department is opened five days a week; patients with epilepsy are appointed once in a week: every Thursday.

### Study population

The study population was all patients with epilepsy who had treatment follow up at the outpatient department within the study period.

Patients aged 18 years and above with a diagnosis of epilepsy and taking treatment at least 6 months were included in the study. Patients with a serious general medical condition and unable to communicate were excluded.

### Sample size and sampling procedures

The sample size was determined using the formula [n = ((Z α/2)^2^ p (1-p))/d^2^], the single population ratio at 95% confidence interval (CI) (Z α/2 = 1.96), and the 5% error margin. Taking the prevalence of the proportion of the population living with epilepsy and suffering from depression from the previous study held in the same region [15] and adding 10% contingency for non-response, the final sample size was 399.

### Operational definitions

#### Anxiety and depression

It was measured by the Hospital Anxiety and Depression Scale [17]. Anxiety and depression were classified as not depressed/ anxious (0–8) and depressed/ anxious (8–21).

#### Social support

It was measured by the Oslo-3 items social support scale (Oslo SSS). The instrument has three items, which has three items with a Likert scale.

A sum index was made by summarizing the raw scores; it ranged from 3 to 14. A score of 3–8 was “poor social support”, 9–11 was “moderate social support” and 12–14 was “strong social support” [18].

#### Perceived stigma

It was measured by 15 item questions with a simple three-point Likert scoring system scored as “not at all” (score of 0), “sometimes” (score of 1) and “always” (score of 2).Then after summing up the result the score above the value on the 66^th^ percentile of the data was defined as the presence of perceived stigma, whereas the score value below on the 66^th^percentile of the data was classified as no perceived stigma [19].

### Data collection and quality control

The data were collected using a pre-tested structured questionnaire, which is developed in English and translated to Amharic then retranslated to English by expertise together with senior psychiatrist to ensure its consistency. The questionnaire was pre-tested on 20 participants at Bahir Dar Felegehiwot referral hospital prior to data collection. To measure the prevalence of depression, Hospital Anxiety and Depression Scale were used, this is a 14 items questionnaire and commonly used to screen for symptoms of anxiety and depression. The 14-items can be separated into two 7-item sub-scales for depression and anxiety. HADS scale was validated in Ethiopia and the internal consistency was 0.78 and 0.76 for depression and anxiety subscales, respectively and 0.87 for the full scale. The scales use a cut-off score for depressions of greater than or equal to 8 [17].

### Data analysis

Descriptive statistic means (SD) and frequency (percentage) were calculated for the continuous and categorical variables, respectively. Scale scores were constructed from items for depression, anxiety, stigma, and social support based on their manuals. After checking chi-square assumption, simple logistic regression was done, and variables with a P-value < 0.2 were selected for the final model. Multiple logistic regression was fitted to assess the association between depression and independent variables. Model assumptions for logistic regression: Hosmer and Lemeshow test were done. A P-value < 0.05 and a 95% confidence interval were used to declare statistically significant. The internal reliability (Cronbach alpha0 of the Amharic version of the HADS was 0.85.The analysis was done with STATA version 14.

### Ethical consideration

Ethical approval was obtained from the Ethical Review Board of the Institute of Public Health, College of Medicine and Health Science, University of Gondar (Ref.No/IPH/180/2019).A permission letter was given to the representatives of the chronic illness OPD. All participants were oriented to the study’s objectives and purpose of the study before they participated, and they provided written informed consent. Patients at health facilities were informed that participation had no impact on the provision of their healthcare and we assure their confidentiality were kept.

## Results

### Socio‑demographic characteristics

A total of 370 participants were interviewed with a 92% response rate. The mean (SD) age of the respondents was 29.7 [11] years. Out of the respondents, more than half (55.4%) were male and the majority of the respondents, 138 (37.3%) had no formal education (Table 1).

**Table 1:** Socio-demographic characteristic of the study participants (n = 370) in Gondar Specialized Hospital, Gondar town, northwest Ethiopia, 2019.

Variables	Frequency (%)
Age (in years)	
18–24	155 (41.89)
25–34	118 (31.89)
35–44	54 (14.59)
>44	43 (11.63)
Sex	
Male	205 (55.41)
Female	165(44.59)
Religion	
Orthodox	339 (91.62)
Muslim	29 (7.84)
Others *	2 (0.54)
Marital status	
Single	195 (52.70)
Married	146 (39.46)
Divorced	16 (4.32)
Others *	13 (3.52)
Educational status	
No formal education	138 (37.3)
Primary education	104 (28.11)
Secondary education	77 (20.81)
College and above	51 (13.78)
Residence	
Rural	185 (50.00)
Urban	185 (50.00)
Occupation	
Formal occupation	217 (58.65)
No formal occupation	153 (41.35)
Wealth index	
Poorest	113(30.53)
Medium	176(47.57)
Rich	81(21.89)

*Others, religion (catholic and protestant); *others, marital status (separated and widowed).

### Clinical and modifying factors

From the total of 370 respondents the majority (61.6%) of them reported that the age of onset for the illness was between 10–25 years. About 42% of them experienced seizure between 1 up to 3 years. While 49.7% of them reported that they are facing stigma as a consequence of their illness (Table 2).

**Table 2:** Clinical and modifying characteristics of the study participants (n = 370) in Gondar Specialized Hospital, Gondar town, Northwest Ethiopia, 2019.

Variables (Description)	Frequency (%)	Mean(SD)
Duration of illness		8.34(6.41)
<5 years	144(38.92%)	
6–10 years	142(38.38%)	
>11 years	84(22.70%)	
Frequency of drug		
Once a day	194 (52.43%)	
Twice a day	144 (38.92%)	
More than twice	32 (8.65%)	
Number of drugs		
Monotherapy	244(65.95%)	
Polytherapy	126(34.05%)	
Frequency of seizure		
Seizure free a year	109(29.46%)	
1–3 year	156 (42.16%)	
More than once in a month	105 (28.38%)	
Adverse reaction		
Yes	57(15.41%)	
No	313(84.59%)	
Comorbidity		
No	345(93.24%)	
Yes	25(6.76%)	
Perceived stigma		9.95(7.82)
Absent	186(50.27%)	
Present	184(49.73%)	
Social support		10.22(2.85)
Poor	97(26.22%)	
Moderate	127(34.32%)	
Strong	146(39.46%)	

### Factors associated with depression

Out of the total of respondents, 37% (95% CI: 32.23–42.09) were found to be depressed. In the final multivariable logistic regression analysis, perceived stigma, educational status, residence, frequency of seizure, and social support were significantly associated with depression.

Those who were stigmatize were 3.89 times more likely to develop depression compared to those who were not stigmatized, (AOR = 3.89, CI: 2.27–6.68). In addition, patients who had poor social supportwere2.73 times more likely to develop depression compared to patients who had strong social support (AOR = 2.73, CI: 1.41–5.31). Whereas patients who achieved primary education levels were 52% less likely to develop depression than patients who were unable to read and write (AOR = 0.48, CI: 0.25–0.92), and patients who live in urban areas were 50% less likely to develop depression compared to their counterparts (AOR = 0.50, CI: 0.28–0.89). Finally, patients who had more than one seizure a month were 2.07times more likely to develop depression compared to patients who were seizure free in a year (AOR = 2.07, CI: 1.01–4.23) (Table 3).

**Table 3:** Multivariable logistic regression analysis of potential factors associated with depression at UoGCSH 2019, (n = 370).

Variables	COR (95%CI)	P-value	AOR (95%CI)
Number of drugs			
Mono therapy	Ref	Ref	Ref
Poly therapy	1.61(1.04–2.50)	0.49	1.22(0.69–2.15)
Perceived stigma			
Absent	Ref	Ref	Ref
Present	4.54(2.87–7.18)	**0.01**	**3.89 (2.27–6.68)** *
Age			
18–24	Ref	Ref	Ref
25–34	0.89(.53–1.49)	0.8	0.91(0.46–1.80)
35–44	1.51(0.80–2.85)	0.42	0.65(0.23–1.84)
>44	3.44(1.70–6.95)	0.55	1.47(0.42–5.12)
Educational status			
No formal education	Ref	Ref	Ref
Primary level	0.36(0.21–0.62)	0.04	**0.48(0.25–0.92)** *
Secondary level	0.38(0.21–0.69)	0.34	0.68(0.30–1.51)
College &above	0.44(0.12–0.52)	0.48	0.71 (0.26–1.87)
Marital status			
Single	Ref	Ref	Ref
Married	1.02(0.65–1.61)	0.55	0.82(0.42–1.59)
Divorced	1.9(0.68–5.32)	0.62	1.39 (0.36–5.54)
Others	10 (2.26–48)	0.27	2.84(0.45–17.84)
Residency			
Rural	Ref	Ref	Ref
Urban	0.53(0.35–0.82)	0.33	**0.5(0.28–0.89)** *
Frequency of seizure			
Seizure free a year	Ref	Ref	Ref
1–3 year	1.67(0.97–2.86)	0.58	1.21 (0.63–2.29)
More than once in a month	2.84(1.6?-5.05)	0.049	**2.01(1.00–4.26)** *
Comorbidity			
No	Ref	Ref	Ref
Yes	2.74(1.2?- 6.29)	0.09	2.32(0.88–6.13)
Frequency of drug			
Once a day	Ref	Ref	Ref
Twice a day	1.78(1.13–2.78)	0.45	1.27(0.7–2.3)
More than twice a day	2.66(1.24–5.68)	0.98	1.01(0.39–2.63)
Social support			
Strong	Ref	Ref	Ref
Moderate	1.74(1.03–2.94)	0.06	1.82(0,98–3.36)
Poor	4.52(2.6?- 7.88)	0.004	**2.73(1.41–5.31)** *
Wealth index			
Poorest	Ref	Ref	Ref
Medium	0.48(0.29–0.78)	0.089	0.59(0.32–1.08)
Richest	0.53(0.29–0.95)	0.053	0.48(0.23–1.01)
Constant			0.19 (0.08–0.44)

*Variables that was significant at P-value < 0.05.

CI, Confidence interval.

## Discussion

In epileptic patients, depression is a common psychological issue that hinders the quality of life and the efficacy of care. The purpose of the study was to determine the prevalence of depression and the related factors in patients with Epilepsy. The findings showed that a remarkable number of patients had depression, and among the many variables, perceived stigma, educational status, residence, seizure frequency and social support were significantly correlated with depression.

In this study, the prevalence of depression was 37% among patients with epilepsy.

This result is consistent with studies at the hospital in Amanuel and in sub-Saharan countries [4,16]. It is, however, lower than studies at Gondar hospital, Illubabaorzone, Jimma hospital, and Black Lion, and far lower than a study conducted in Nigeria [1,8,10,13,15]. The results is also higher than those of studies carried out in Jordan [9]. Such inconsistencies can be due to the variations in instruments for assessing depression.

The current study found that among patients with perceived stigma, depression was greater than those without perceived stigma, which is comparable to a study conducted at Amanuel Hospital and Black Lion Hospital [1,16]. In those who have repeated seizures, however, depression has increased relative to those with lower seizure experience. This finding is in line with studies Amanuel Hospital, Gondar Hospital, Illubabor ZoneHospital and Jimma Hospital [10,13,15,16].

In this study, the risk of depression among patients with lower educational status was lower than those with higher educational status. This correlation was consistent with a study at Jimma Hospital [13]. However, this finding contrast with studies from hospitals in the Gondar hospital and Illubabor district [10,15]. This may be because, by chance, participants with high educational levels were poor in our sample. Compared to patients with good social support, this study found that depression was higher among patients with weak social support. This is in line with a report at hospitals in Illubabor [10] the possible explanation might be having good social support decreases stress and tension on the disease.

In the current research, the risk of depression among urban dwellers was lower than for those living in rural areas. The potential reason may be that patients in urban areas could have better access to health care services and receive advice from health professionals.

The study was decided by self-reports, the drawback of the current study is that social desirability bias could be there because other approaches could not validate the tools. In fact, this could under estimate the degree of the depression.

The presence of higher numbers of non-respondents could underestimate or overestimate the magnitude of depression due to the nature of the disease most of the participants lack of willingness to participate. In addition recall bias due to failure to remember long-term history and using cross-sectional study design only estimate the prevalence at a point might underestimate or overestimated the prevalence.

## Conclusion

At Gondar Specialized Hospital, the prevalence of epileptic patients who were found to be depressed was high. The presence of perceived stigma, primary education, urban residents, high seizure frequency and inadequate social support were significantly correlated with patients’ depression. Health care professionals should give more attention to people with perceived stigma, a having frequent seizures, and patients with low social support levels.

## Supporting information

S1 File Stata data for depression. (DTA)

## Abbreviations

BDI: Becks Depression Index
HADS: Hospital Anxiety Depression Scale
PWE: People with Epilepsy
UoGCSH: University of Gondar Comprehensive and Specialized Hospital
